# As Steady as a Rock! Gaining Insight in Recruitment and Retention Among Primary School Children With Behavioural Problems in Sport Mix Club

**DOI:** 10.3389/fpubh.2021.547634

**Published:** 2022-01-11

**Authors:** Lotte Prevo, Maria Jansen, Dave Van Kann, Stef Kremers

**Affiliations:** ^1^School of Nutrition and Translational Research in Metabolism, Department of Health Promotion, Maastricht University, Maastricht, Netherlands; ^2^Department of Health Services Research, Maastricht University, Maastricht, Netherlands; ^3^Academic Collaborative Center for Public Health, Public Health Service Southern Limburg, Heerlen, Netherlands; ^4^School of Sport Studies, Fontys University of Applied Sciences, Eindhoven, Netherlands

**Keywords:** behavioural problems, recruitment, retention, self-determination theory, primary school children

## Abstract

The number of children dealing with behavioural problems is increasing. A major challenge in many health-supportive programmes is the recruitment and retention of these children. In the current study, Sport Mix Club (SMC), an approach to enhance socioemotional disorders of 4- to 12-year-old children through sport classes in municipality Vaals, the Netherlands, is used as an illustration. Where many studies faced difficulties getting and keeping children in their interventions, SMC overcame this challenge. Therefore, we decided to explore “What factors contribute to enhanced recruitment and retention procedures among children with behavioural problems in Sport Mix Club?” A qualitative case study design using the analysis of the administrative logbook of the SMC coach and trainees, individual interviews with the SMC coach, trainees (*n* = 2), school teachers (*n* = 3) and parents of participating children (*n* = 9), and four focus group interviews with children (*n* = 13) were carried out. During the recruitment and retention of SMC, the human psychological need of relatedness seemed to be of crucial value. The fact that the SMC coach: (1) made efforts to become a familiar face for children, parents and community partners beforehand; (2) showed enthusiasm; and (3) placed her focus on having fun as opposed to the children's problems, seemed to be decisive in the process of getting children to participate in SMC and retaining their participation.

## Introduction

Children having trouble concentrating, acting as the “rebel” in class or being so shy that it makes it difficult for them to make good connexions, are some examples of behavioural problems that teachers come across in the classroom situation. Behavioural problems in children are defined as externalising behaviour, e.g., demonstrating disruptive, rebellious, antisocial or aggressive behaviour, and as internalising behaviours, e.g., being anxious, worrying excessively or unable to make contact (1). The number of children in need of extra support due to their behavioural problems and the lack of support from their environment is rising, especially in most western countries, where nearly 20% of the children and adolescents are dealing with behaviour problems (2). In the Netherlands for example, the prevalence increased from 9.7% (243.000 children) in 2003 to an estimation of over 12% (about 300,000 children) in 2018 (3). These problems often arise from a lack in their social and emotional development (4). Behavioural problems have been found to lead to lower school performances (5) and therefore have a negative influence on future outcomes such as income, career perspectives, well-being and health (6). Timely support for this group of children is expected to enhance their functioning not only at school, but also at home and within the broader community.

Studies have shown that poverty combined with a low socioeconomic status (SES) in the home environment accelerates the development of these problems, making these children more vulnerable (7, 8). Parents of low-SES families were found to be less responsive to their children's needs and were less able to relate more interactively with their children to support their socioemotional development (9, 10). It is important to consider that due to the circumstances in which low-SES families live, parents may have developed a tunnel vision based on income scarcity. This means that they are likely to devote most of their mental capacity to their daily issues, making them unable to act upon other goals, such as the socioemotional development of their child (11, 12). Furthermore, in schools, teachers seemed to have lower academic expectations concerning children from a lower-SES family than for children with a moderate to high SES background who likely lived in a more supportive home environment (13).

Various approaches are in place to timely support children with behavioural problems, focusing, for example, on the parenting skills at home and training the teachers at school to ensure that the environment is optimised, and providing socioemotional skills training for the individual child (2, 14–16). Although these approaches are intended to support children with behavioural problems, their impact is limited when the target group cannot be reached. The main challenge remains to recruit the most vulnerable children, i.e., those with low socioeconomic status and in poverty. Then we need to sustain their participation in the whole programme. Although various recruitment and retention strategies are used, the effects of these strategies produce mixed results, leading to few successful examples (17). Therefore, more rigorous research is needed in defining factors explaining the recruitment and retention success.

It is clear that all existing and new approaches face the challenge of tailoring the recruitment procedure to approach and attract the difficult-to-reach participants to participate in health and well-being supportive programmes and ultimately sustain participation (18). For children, the recruitment procedure frequently uses informative messages that are normally spread via school to their parents. These messages quite often include detailed information about the programme itself, but insufficiently touch upon the needs experienced by families facing challenges that accompany a low-SES status. Besides, parents must approve participation, which in practise often means that the low-SES children are not reached due to the high mental load of the parents that keeps them from letting their children join a programme. To actually reach these children, recruitment procedures need to be considered that highlight the personally relevant needs of low-SES parents and their children to ensure that they see the added value and become motivated to let their child participate (19). This approach, where we focus at enhancing intrinsic motivation is also explained in the Self-Determination Theory (SDT) (20). This theory assumes that when the basic psychological needs for autonomy (= being in charge of own action), competence (= mastering an activity) and relatedness (= being connected to relevant others) (20) of both parents and children can be satisfied, their quality of motivation to participate can grow, increasing the chance of improved recruitment and retention (20).

A best-practise example on recruitment and retention in a low-SES community is Sport Mix Club (SMC). Within this approach, primary school children with behavioural problems are offered support with their socioemotional competences. By using sport classes as a means, children are expected to develop these competences so that they are better able to participate within the school, home and community context (21). The successful recruitment and retention within this SMC approach made us consider exploring contributory factors for these results in more detail. These factors are expected to touch upon the basic needs and quality of motivation for both parents and their children in accordance with the principles of the SDT (20). Results could be helpful in strengthening recruitment and retention methods for populations defined as hard-to-reach. The current study focuses on the research question: “What factors contribute to enhanced recruitment and retention procedures among children with behavioural problems in Sport Mix Club?”

## Methods

### Study Design

We carried out a qualitative case study, using a constructivist paradigm to focus on the manifold views, experiences and interpretations of SMC coaches and participating children, their parents and primary school teachers with regard to SMC. Within the constructivist paradigm, the gathered data in relation to the recruitment and retention strategies of SMC were analysed to gain a better understanding of this complex phenomenon. The case study design was expected to be helpful in generating in-depth understanding of the recruitment and retention strategies of SMC in a real-life practical context. We started with an inductive process to understand the data, followed by an abductive process to define the possible explanations for successful recruitment and retention using relevant theoretical background (22, 23).

### SMC Approach

SMC aimed to support primary school children in dealing with behavioural problems through sport classes. During the intake, the motor and socioemotional competences of the participating children were screened by the SMC coach. Children with behavioural problems are encouraged to improve their motor and socioemotional competences, since this was found to be beneficial for their daily functioning (14). In small groups with a maximum of twelve primary school children, different sports activities are experienced with the support of coaches, once a week. By using an element of fun, exercising in groups and individual support from the coaches, the focus lies on transcending factors, such as self-esteem, problem solving, taking initiative, independence and perseverance, with the aim of developing a more positive self-image. The SMC approach specifically focuses on children discovering their own talent so that the children get a boost in self-worth and become aware of their own capabilities. The sports activities had a non-competitive nature to avoid situations focused on emotional profit or loss. Since SMC focused on long-term participation without defining a specific endpoint, retention was seen as a challenge. During the research, we observed that the SMC approach is theoretically based on the basic psychological human needs of the SDT, indicating that it may include the possibility for the children to control the outcome of the activity and master it (competence), while having connexions with relevant others, feeling a sense of belongingness within the sport classes (relatedness), and overall having the feeling of being the agent of their own actions (autonomy) (20).

### Study Setting

The current study was executed in the municipality of Vaals, the southernmost part of the Netherlands, in the period from March 2019 till May 2019. In the age group from four to twelve years, Vaals has about 500 residents (24). One in eight youngsters (12.5%) of the youth aged below 23 years, including children with behavioural problems, receives specialised youth care; this is higher than the Dutch average of about 10% (25). Based on this percentage, we expected that about 50–55 primary school children have behavioural problems in Vaals. Furthermore, the municipality is defined as having a moderate- to low-SES and can often be found in the top ten municipalities experiencing poverty (26, 27). SMC is operating within a population that can be described as very hard to reach. In total, three primary schools are located within the municipality, together accounting for 450 children.

### Study Participants and Recruitment

From March 2019 till May 2019, fifteen individual interviews and four focus group interviews were carried out. The interviews were held with the coach of SMC, two trainees, three teachers of the schools in Vaals who fulfilled the role of gatekeeper for the SMC coach, and nine parents of children taking part in the programme. The teachers were contacted via email, providing them the information needed to decide on whether or not to participate in the study. The researcher (LP) visited the SMC classes to inform parents about the study and to answer their questions. Thereafter, parents were recruited face-to-face via the SMC coach. Since not all parents were present in the classes, an information letter was sent per email as well. Parents could either directly confirm to the researcher or SMC coach if they wished to take part in the interviews or could respond to the coach's email. Additionally, focus group interviews were conducted with a total of 13 children, aged between six and twelve. To recruit the children for the focus group interviews, the researcher visited the SMC classes and explained the study to the children. Afterwards, all children received an information letter and informed consent form for their parents to sign on paper, which they had to bring to the SMC class within two weeks.

### Data Collection and Instruments

First, the 30–45 min individual interviews used semi-structured questionnaires and were audio recorded. Before each individual interview, the procedure was explained, and the informed consent form was signed. For the individual interviews with coaches, trainees, teachers and parents, slightly different questions were asked, focusing on relevant aspects of SMC for the specific groups (Table 1). The trainees and SMC coach were specifically asked to explain their role within SMC, and describe the content, purpose and recruitment and retention of SMC. Parents were asked to say how long their child had been participating in SMC, why they were participating, how they were informed about the possibility for their child to take part in SMC, and whether the child appreciated the SMC classes. Teachers were asked about their motivation for referring children to SMC. In all the individual interviews, the researcher asked questions about possible changes in behaviours and well-being of the children, their overall opinion about SMC, and whether they saw any opportunities to further improve SMC. SDT concepts and questions related to the recruitment procedure and retention were not explicitly mentioned so that participants could express their views, experiences and interpretations about the SMC approach without too much guidance from the researcher. During the individual interviews the basic human needs of the SDT were taken into consideration, while interviewees answered the more open formulated questions. We selected this approach to ensure the interviewees to share their own experience without focusing too much at these needs in the first place.

**Table 1:** Semi-structured interview guides.

Individual interview with SMC coach - How do you define your role within SMC? - What does the SMC programme entail? - What is the purpose of SMC? - There are some children who need more support, how do you ensure they participate? - Which developments do you notice in the behaviour and well-being of the participating children? - How would you like to enhance the SMC approach?
Individual interview with SMC-trainees - What is your role within SMC? - What does the SMC programme entail? - Which developments do you notice in the behaviour and well-being of the participating children? - How can we further improve SMC?
Individual interview with teachers - How many students have you referred to SMC? - Why did you refer these students to SMC? - Do you notice some changes in behaviour/well-being of these children in the classroom? - Has SMC fulfilled your expectations as to what you hoped to accomplish with the referral of the children to SMC? - How can we further improve SMC? - How do you see the role of the SMC-coach in general?
Individual interview with parents - How long has your child been participating in SMC? - What is the reason of participation for your child? - How does your child like going to SMC? - Do you notice changes in your child's behaviour/well-being? If so, which ones? - How can we further improve SMC?
Focus group interview with children - Start with a drawing or Post-it Notes. - Round 1 – where all children explained what happened in their drawing or explained what they had written down. Followed by together deciding on the themes discussed. - Round 2 – all themes were written down by the researcher during the session to prioritise together with the children in the focus group interview.

Second, prior to each focus group interview, the semi-structured procedure was explained and the recording was started. During the focus group interviews of about one h, children were asked to make a drawing of SMC or write down words about SMC on Post-it Notes, helping the children's voices to be heard. This could be about anything that was related to SMC in their eyes, e.g., what they liked or disliked or what they had learned. After about 10 min, the drawings and Post-it Notes were discussed, with each child being asked in turn to explain what they had drawn or written about the SMC classes. We received eleven drawings from the children aged ten years old or younger and two posters with mainly post-its from the older children. The most important themes that arose were selected by the children. During the final individual interviews and focus group interviews, no new information was retrieved, indicating data saturation and confirming to us that we had completed the data gathering process. Although the principles of the SDT were taken into consideration during all interviews, we decided to use an open format, without explicitly asking for all basic human needs. We started each focus group by asking children to draw or write down what was important to them in relation to SMC. During the group discussion the basic human needs were discussed, without explicitly mentioning them, when children came up with their themes.

Finally, we had access to the coach's administrative logbook containing anonymized information about the participating children for background information. The administration contains information on age, gender, class in school, referred/subscribed by, reason for participation, start date and, if applicable, end date with outflow information.

### Data Processing and Analysis

All individual interviews and focus group interviews were recorded and transcribed verbatim. To correctly organise the data to interpret recruitment and retention methods within SMC, the Nvivo version 12 pro software was used (28). The analysis started with open coding, where the codes were related to the data gathered in the interviews. We aimed to stay very close to the information interviewees provided for example codes related to the role of the SMC coach e.g., “*visible in school”* and “*familiar face*,” the experiences of the children and parents with SMC and the coach e.g., “*fun”* and “*enthusiastic,”* and some developments of children during SMC e.g., “*proud of progress”* and “*opportunities for personal preferences*.” Based on these raw codes, related to the interview questions in Table 1 for each group, we started with the next step of axial coding. The codes were refined and connexions were drawn between the codes. In this phase, we already clustered all individual codes to find an overarching pattern. The SDT seemed to be very useful, so we already aimed to cluster more toward autonomy, competence and relatedness in this phase. Finally, selective coding was used to make central categories of the information participants mentioned about recruitment and retention, i.e., in particular related to autonomy, competence and relatedness as described in the second phase of axial coding. In the final phase, we ended up with two main mother codes focused on recruitment and retention and for each mother code the three subcodes of autonomy competence and relatedness for all groups of participants: the children, parents, teachers, trainees and the coach. To sum up, the raw codes were eventually recoded as one of the three basic needs theoretically related to SDT for both recruitment and retention and agreed upon by the project team members (29). To analyse the administrative logbook, the variables were imputed into SPSS IBM statistics version 23 enabling the researcher to make an overview of all defined background characteristics in the descriptive statistics software.

## Results

### Background Information of Participating Children

SMC was initiated in September 2017. During the almost two years that the approach has been available in Vaals, 28 children have participated. Compared to the total number of children with behavioural problems, SMC succeeded in recruiting about half of all eligible children in this hard-to-reach population. The majority of participating children (*n* = 24) took part in SMC for a year or longer. When starting, about half of the children were aged ten or above (57%). The schoolteachers formed an important gatekeeper since the majority of the participating children had been referred by them (56%). Other children were mainly directly put forward by their parents without the teacher as intermediary. In the focus groups, a total of thirteen children participated within the age range of six till twelve, with the majority of children aged above nine.

*Trainee: “You really notice that the children for whom SMC is intended are actually here. I think that is important, because if the objective is to attract and support children with a lack of motor and social competences, they must also be here and they clearly are.”*

### Recruitment

The role of the SMC coach during the recruitment procedure was described as connecting and motivating during all individual interviews and focus groups, focusing at a strengthened relationship with local partners, children and parents.

Since 2012, the SMC coach has carried out several activities at school and in her work as a community youth worker. The fact that the SMC coach was present in these settings for a relatively long time period was said to make her a familiar face among the children and parents. She indicated the importance of staying visible and helpful, and described it as a necessity to keep the trust that children, parents and teachers have in her. The involvement of the coach was appreciated by teachers and parents. The SMC coach knew the children well and could discuss her findings during the regular sport classes directly with the teachers. In general, children and parents mentioned to end up in trusted “familiar hands,” something that the teachers and coach also stated to be important.

*SMC coach: “I think it is important for a coach to be in schools and in the neighbourhood. That is how children, parents and partners get to know you and how you create a sufficient level of trust. Just “being there” is necessary to get things done.”*

With regard to the motivating role of the SMC coach during the recruitment for SMC, the enormous amount of energy, the high level of enthusiasm, the accessible way of informing teachers and parents about SMC, and the positive mindset of the SMC coach were mentioned during almost every individual interview or focus group interview. The element of “having fun” and the focus on discovering the talents of the children particularly stood out, while the focus on the lack of competences of the child was underexposed or not even mentioned by the SMC coach during recruitment. This message was regarded by some parents as the reason to find it rather hard not to get enthusiastic about SMC.

*Teacher: “What I like a lot is that the coach is someone who enters the school once in a while. She is very involved and the children know her well.”*

*Parent: “The coach is worth her weight in gold. I am always thinking, just find someone who carries out all those activities and shows so much enthusiasm. It is contagious. If you are in her presence for just five min, then you have the idea that you have to do something too.”*

As a point requiring improvement, teachers and the coach explained that children may be referred earlier to SMC. They explained that the younger a child started with the approach, the easier it was to make progress. Additionally, since SMC practises ongoing recruitment, professional partners working in the community or at school may also be kept informed about SMC, enabling them to continue to refer children.

*Teacher: “What I notice is that we as teachers still wait too long before we refer a child to SMC. Especially, with the younger children, the issues are not so clear yet and we tend to wait to see how a child develops. It would be better to ask the SMC coach sooner for advice to find out if a particular child can benefit from the SMC approach.”*

### Retention

After participating in SMC, the large majority of children and their parents stated that children enjoyed the classes. It was mentioned that the coach played an important role in creating this pleasurable environment where children got the opportunity to develop. Parents said that the coach always had a positive approach toward the children. They never heard her speaking negatively and she was very enthusiastic. This was explained as being motivating for their children to continue to attend. The relatively large variation in the SMC activities was mainly regarded by parents and children as motivating. The coach also expressed paying extra attention to the social aspect to make every child feel safe and comfortable in the class. The personal attention that was given by the SMC coach by engaging with a child separately if necessary was also mentioned by the parents and teachers. Parents said they were sufficiently informed by the coach regarding the progress of their child and if any problems arose these were discussed and acted upon immediately. Additionally, it was stated that all children had their own issues, resulting in no child standing out when some extra support was given. Children said that they had more fun when they did the activities together. The support of the coach and trainees was said to be important, as well as the cooperation with other children supporting them to become better. The children and parents mentioned a strengthened sense of belongingness within the role of the SMC coach. Teachers explained that this sense of belongingness was an important advantage compared to regular educational practises, where they had less time for individual children.

*Parent: “They involve everyone in the classes. Recently, they did a breakdance workshop. They all stood together in a circle and could show what they had learned to the others.”*

*Parent: “The feedback of the coach is very good. If something happens, she will also come here and tell the parents that someone is feeling sad for a moment, or does not want to participate for a second.”*

While gaining insight in the retention of children in SMC, the importance of relatedness was frequently mentioned in both individual interviews and focus groups. Additionally, children in the higher classes of primary school explained that they liked some level of freedom in SMC. Children could make suggestions about the content of the classes, and within certain activities they were given some freedom to carry out the activity the way they wanted. Parents mentioned this as well as the opportunity that children were given to do their own thing for a moment. This indicates that autonomy was integrated in the SMC approach as well. Furthermore, all children themselves, their parents, teachers, and the coach mentioned that children had developed both their motor and socioemotional competences, e.g., climbing higher, running faster, dealing with tasks that were difficult before and working together with other children. Children mentioned that they were proud about what they had learned. They valued the good feeling it gave them when they became better at something that was previously difficult. Thereby showing that enhancing competence was also in play during SMC classes.

*Child (autonomy): “You can act freely with the monkey cages activity. Then you can be yourself. With the running and stuff you can choose which path to take and you do not have to think about having to do this or that.”*

*Child (competence): “I think climbing from the closet into the rope is pretty exciting, but when I managed to do that, it felt really good.”* *Child (relatedness): description of a drawing “Yes… those are the ropes and children climbing them. This is the SMC coach; she also climbs the ropes together with us.”*

## Discussion

The current study aimed to explore crucial factors for both the recruitment and retention of primary school children with behavioural problems in the SMC approach. The factors participants mentioned in relation to recruitment and retention were matched to the human psychological needs, autonomy, competence, and relatedness of the SDT to find out what motivated parents and children to take part in SMC and continue participation. During the recruitment procedure, results showed the focus of all participants on the human psychological need of relatedness between children and their SMC coach. The SMC coach seemed to be able to touch and engage relevant others closely related to the child and demonstrated the specific interpersonal characteristics needed. For seven years, the SMC coach has been actively involved within the community to strengthen collective activities for children and youth based on physical activity, but also their broader social development. During this period, the SMC coach created connexions with community partners who have close contact with (vulnerable) children and their parents, e.g., the schools and teachers. This also made her a familiar face for parents and children living in the community. The relatively long time period that the SMC coach has been active, described as the “simmering time,” provided SMC with an advantage before the actual recruitment procedure for SMC took off. As mentioned by Bartlett et al. (30) partnering with schools was found to be beneficial in recruiting school-age children for programmes or research. They found that by engaging formal assistance from the school, suspicions among children and their parents decreased. The trust of children and parents within the schools was found beneficial for recruitment (31). This was even described to be more successful if a member of the school staff assisted with the recruitment. The latter also seemed to be of greater value to underserved groups, e.g., low-SES populations (32). Although the SMC coach was officially not a member of the school staff, the fact that she was highly involved in the school, had the expertise to timely indicate children in need of extra support, stayed visible and developed herself as a familiar face to teachers, parents and children seemed to have made her a trusted person within the school (33). Additionally, the SMC coach did not only recruit the children at the schools, she also implemented SMC immediately after school hours, ensuring that children continued to be supported by the same trusted person, making her as steady as a rock (34).

Specific interpersonal characteristics of the SMC coach in combination with the message she spread also seemed to contribute to the recruitment. The coach was described as having an enormous amount of energy, a high level of enthusiasm and a positive focus. The SMC coach seemed to have the necessary interpersonal characteristics to be successful, since she was both energetic and passionate about her work, which seemed to encourage children and parents to participate. As a recruiter, the strength seemed to be in the power to motivate parents and children, with a high level of integrity and reliability. In addition, the communication skills needed to spread the message seemed to be a perfect fit with the target group. As described in the work of Ellard-Grey et al. (35) labelling the population was seen as an extra challenge for people in a more vulnerable situation, especially because these populations may perceive a level of stigmatisation when participating in a programme such as SMC. The explicit message of the SMC coach did not focus at all on the behavioural problems of the children and their socioeconomic difficulties, but on the opportunity for children to discover their talents for a specific sport. By making the element of fun a key factor while exercising and presenting SMC as just a regular sports activity, the stigmatisation risk decreased. The important thing was that the SMC coach had sufficient expertise to use some screening to prevent non-intended populations being included. Overall, the fact that parents and children felt a high level of relatedness to the SMC coach and her approach in SMC, turned out to fulfil a very important basic human need. The satisfaction of this need for relatedness seemed “the key” to motivate parents and children to start in SMC.

The optimised learning environment created by the SMC coach seemed to have “having fun together” as a key element. It showed that the SMC coach had job-related qualifications that may have enhanced retention. Especially with regard to programmes where sports were used, enjoyment was described as an important driver for retention among children (36–38). The variety of activities, maintaining a sufficient level of novelty, and the positive, enthusiastic, and motivating approach used by the SMC coach were said to support the social aspect, ensuring that every child felt safe and comfortable in the class. Especially for retention, McCann et al. (39) stressed the necessity of a suitable coach, with job-related qualifications such as (feedback) skills and having an appeal for the parents and children. A coach should have the ability to engage participants and tailor the programme toward them, acting as a programme champion (39). A strong focus on the human psychological need of relatedness became evident for retention as well. The personal attention for children and their parents that the SMC coach provided seemed to develop and sustain the relationship based on caring, trust and respect (40). The work of Anderson-Butcher (34) showed that a level of social approval by the coach with timely feedback was used to reinforce their involvement. The SMC coach also focused on creating peer friendships via their participation, not only between the children, but also among their parents, which may also provide opportunities for shared and cooperative learning (34, 41).

In addition to the basic human psychological need for relatedness, the needs for autonomy and competence, e.g., having a say and responsibility balanced with structure and consistency, were also described as being important for enhancing retention. In the work of Deci and Ryan (20) and Reeve and Jang (42), it was indicated that the quality of motivation and level of having fun improved when children were given some level of autonomy, as it provided them with a level of control over their environment (34). The psychological human need of competence, especially the improvement of motor and socioemotional competences, was valued by children. A balance between failures and successes was integrated in SMC for children to gradually improve by applying effort. Especially the growth in what children had learned made them feel proud, resulting in an adequate level of perceived satisfaction (34, 43). As expected, the three basic human needs for autonomy, competence, and relatedness were approximately equally important to explain why parents and children continued participating in SMC. As the SDT assumes, all three needs are important to strengthen levels of intrinsic motivation to continue participating (20).

In conclusion, the current study showed that successful recruitment and retention were strongly related to the basic human psychological needs of SDT, in combination with interpersonal characteristics and job-related qualifications of the coach.

### Strengths and Limitations

A major strength of this study was the good representation of the hard-to-reach target group. It is interesting that children aged ten or above seem to drop out of sports participation, while in SMC this group was recruited most (44). Furthermore, the inclusion of different data sources and the various views, from the SMC coach, trainees, schoolteachers, parents and the children, were helpful in creating an in-depth and complete overview of the value of the SMC approach, and more specifically on the successful recruitment and retention within the approach. The interactive techniques of letting children make a drawing or use Post-it Notes was helpful as well as enabling them to express their thoughts and opinions about SMC.

In addition to these strengths, some limitations should be acknowledged. First, SMC has been implemented in only one municipality, making the number of participating children and thereby the number of individual interviews and focus group interviews limited, which might have resulted in insufficient information. Second, although a diverse group of children and parents participated, the necessity to sign an informed consent may have discouraged parents from letting their children participate or from participating themselves in the current study. Finally, it is possible that when the human needs within the SDT, autonomy, relatedness and competence, were raised more specifically during the interviews, interviewees would have had more to say about SMC in that sense. Although the theoretical framework and the focus on the needs were not included before starting the interviews, the abductive process taken afterwards was helpful to define the possible explanations for successful recruitment and retention within the SDT. Therefore, we still believe that we gathered all relevant data to answer our research question. The more open data gathering process, without the focus on specific theoretical insights, might even helped interviewed parents and children to express themselves. These theories are often more difficult to understand for lay people, which indicates that it might not be an issue that our study did not start from a specific theory, but that the theory was included during the research process (10).

### Recommendations for Future Research and Practise

The first qualitative results about recruitment and retention in the SMC approach turned out to be promising. The exploration of factors found in this study that may explain this success in recruitment and retention can be enhanced by more quantitative research with regard to the implementation process of this kind of approach and should be scaled up to other regions for further generalizability. With regard to practise, especially, building on the relatedness, where the SMC coach can become a familiar, trusted face in school and in the community, requires time, which should be provided. A role for teachers in which they assist the recruiters and express the benefits to children and parents of a specific approach would be beneficial to make underserved groups participate. Further, interpersonal characteristics seem to be extremely relevant for successful recruitment, whereas job-related qualifications seem to be important to retain children in health-supportive programmes such as SMC. The development of both competences need further attention in educational programmes for sports coaches and other professionals working with vulnerable groups to become sensitive to the needs of their target group (45).

## Data Availability Statement

The original contributions presented in the study are included in the article/supplementary material, further inquiries can be directed to the corresponding author/s.

## Ethics Statement

This application to the FHML-REC was made in relation to project work that had already been undertaken. Therefore, the committee cannot approve the work in advance. The FHML-REC will not give retrospective approval for any work after it has been undertaken. It should be noted that the reason that the project did not obtain approval in advance of the work was because in the Netherlands only certain projects involving humans are required by law to be reviewed by an ethics committee. Researchers undertaking work outside that requirement had no committee to approach to assess the ethics of their work. Recently, Maastricht University's Faculty of Health, Medicine and Life Sciences has created the Faculty Research Ethics Committee (FHML-REC) to address this shortcoming. This project fell into this category - it started before a committee or assessment was available. The FHML-REC has now reviewed the work that was undertaken. It found that the work was undertaken in a manner that conformed to the current ethics standards of Maastricht University. The Data Protection aspects of the work were reviewed by the Faculty AVG/ Data Protection team and were found to be compliant with the requirements. Your reference number is: FHML-REC/2020/070. Written informed consent to participate in this study was provided by the participants' legal guardian/next of kin.

## Author Contributions

LP, MJ, DV, and SK contributed conception and design of the study. LP gathered and analysed the data and wrote the first draft of the manuscript. MJ, DV, and SK critically reviewed and revised sections of the manuscript. All authors contributed to the article and approved the submitted version.

## Funding

This work was supported by FNO (Fonds NutsOhra) (Grant 31963254N).

## Conflict of Interest

The authors declare that the research was conducted in the absence of any commercial or financial relationships that could be construed as a potential conflict of interest.

## Publisher's Note

All claims expressed in this article are solely those of the authors and do not necessarily represent those of their affiliated organizations, or those of the publisher, the editors and the reviewers. Any product that may be evaluated in this article, or claim that may be made by its manufacturer, is not guaranteed or endorsed by the publisher.
